# Peroxisome Proliferator Activated Receptor-**α** Agonist Slows the Progression of Hypertension, Attenuates Plasma Interleukin-6 Levels and Renal Inflammatory Markers in Angiotensin II Infused Mice

**DOI:** 10.1155/2012/645969

**Published:** 2012-07-16

**Authors:** Justin L. Wilson, Rong Duan, Ahmed El-Marakby, Abdulmohsin Alhashim, Dexter L. Lee

**Affiliations:** ^1^Department of Physiology and Biophysics, College of Medicine, Howard University, 520 W Street NW, Washington, DC 20059, USA; ^2^Department of Oral Biology and Department of Pharmacology and Toxicology, Georgia Health Sciences University, Augusta, GA 30912, USA

## Abstract

The anti-inflammatory properties of PPAR-**α** plays an important role in attenuating hypertension. The current study determines the anti-hypertensive and anti-inflammatory role of PPAR-**α** agonist during a slow-pressor dose of Ang II (400 ng/kg/min). Ten to twelve week old male PPAR-**α** KO mice and their WT controls were implanted with telemetry devices and infused with Ang II for 12 days. On day 12 of Ang II infusion, MAP was elevated in PPAR-**α** KO mice compared to WT (161 ± 4 mmHg versus 145 ± 4 mmHg) and fenofibrate (145 mg/kg/day) reduced MAP in WT + Ang II mice (134 ± 7 mmHg). Plasma IL-6 levels were higher in PPAR-**α** KO mice on day 12 of Ang II infusion (30 ± 4 versus 8 ± 2 pg/mL) and fenofibrate reduced plasma IL-6 in Ang II-treated WT mice (10 ± 3 pg/mL). Fenofibrate increased renal expression of CYP4A, restored renal CYP2J expression, reduced the elevation in renal ICAM-1, MCP-1 and COX-2 in WT + Ang II mice. Our results demonstrate that activation of PPAR-**α** attenuates Ang II-induced hypertension through up-regulation of CYP4A and CYP2J and an attenuation of inflammatory markers such as plasma IL-6, renal MCP-1, renal expression of ICAM-1 and COX-2.

## 1. Introduction

 Peroxisome proliferator-activated receptors (PPARs) are nuclear hormone receptors that have attracted enormous attention during inflammation and blood pressure regulation [1–3]. Peroxisome proliferator-activated receptor alpha (PPAR-*α*) ligands negatively regulate interleukin-6 (IL-6) promoter activation [4], and chronic treatment with fenofibrate, a PPAR-*α* agonist, suppresses IL-6-induced mechanisms [5]. Devchand et al. [6] demonstrated that the absence of PPAR-*α* expression in mice prolonged the inflammatory responses, suggesting that PPAR-*α* has anti-inflammatory properties [7, 8]. Previous reports suggest that the PPAR-*α* ligand, fenofibrate, represses ICAM-1 [9] in mouse cardiac tissue and VCAM-1 [10] expression in endothelial cells.

In addition to the anti-inflammatory effects of PPAR-*α* agonists, recent reports also demonstrate that PPAR-*α* ligands decrease blood pressure in various models of hypertension [11–13]. Several mechanisms have been proposed to explain the antihypertensive effects of PPAR-*α* agonists such as increased excretion of Na^+^ through reduced Na^+^-K^+^ ATPase activity in the proximal tubule [14], increased cytochrome P450 4A (CYP4A) expression [15], and increased renal tubular 20-HETE production [12], which exerts a natriuretic effect [11, 16]. A recent report from our laboratory demonstrates that the PPAR-*α* agonist fenofibrate decreases mean arterial pressure and plasma IL-6 during an acute model of DOCA-salt hypertension [17], suggesting that there is a crosstalk between PPAR-*α* and IL-6 in regulating blood pressure.

 Angiotensin II (Ang II) stimulates proinflammatory cytokines and downregulates cytochrome P450 (CYP)-derived metabolites of arachidonic acid (AA), which are important regulators of renal vascular tone and tubular function [19]. Angiotensin II (Ang II) stimulates the release of IL-6 [20–23], and a previous report demonstrates that Ang II hypertension is attenuated in IL-6 knockout mice [24]. Ang II also upregulates many proinflammatory genes, such as intracellular adhesion molecule-1 (ICAM-1) and monocyte chemoattractant protein-1 (MCP-1), through the activation of several intracellular signaling mechanisms, including nuclear factor-*κβ* (NF-*κβ*) [25]. Cyclooxgenase-2 (COX-2) is considered a cytokine-induced cyclooxygenase [26–28], as NF-*κβ* has been shown to increase COX-2 in the kidney [29], and Ang II has also been shown to stimulate glomerular COX-2 protein expression [30]. Ang II reduces CYP4A expression and renal tubular 20-HETE, whereas stimulation of PPAR-*α* with fenofibrate causes a significant increase in both CYP4A and 20-HETE production during Ang II hypertension [12]. In addition to the antihypertensive effect of tubular 20-HETE, epoxyeicosatrienoic acids (EETs) are the major vasodilator arachidonic acid metabolites of cytochrome *P*450 epoxygenase enzymes. EETs are synthesized predominantly by the epoxygenases of the CYP450 family, including the 2C and 2J classes [31], fenofibrate has been also shown to increase renal hydroxylase enzyme as well as epoxygenase protein expression [32]. Decreased CYP2J expression is associated with impaired mesenteric artery relaxation and also is reported in the renal vasculature of animals treated with Ang II plus a high-salt diet [33, 34]. The goal of the current study is to determine whether PPAR-*α* activation during a slow pressor dose of Ang II would decrease proinflammatory mechanisms involving plasma IL-6, renal MCP-1, renal expression of ICAM-1 and COX-2, while also stimulating the expression of CYP4A and CYP2J to attenuate increases in blood pressure.

## 2. Methods

Procedures involving animals were approved by the Howard University Institutional Animal Care and Use Committee. Blood pressure transmitters (Data Science, PA-C10, St. Paul, MN, USA) were implanted in age and weight matched (10–12 weeks, 25–28 grams) male PPAR-*α* knockout (−/−) (B129S4/SvJae-*Ppara* ^*tm*1*Gonz*^/J), and wildtype (+/+) mice (B129S1/SvImJ) from Jackson Laboratories (Bar Harbor, ME, USA). The animals were anesthetized using isoflurane and biotelemetry transmitter devices were implanted using aseptic techniques. The catheter was implanted in the left carotid artery through an incision in the vessel wall made with a custom-shaped 27.5 gauge needle. The body of the transmitter was tunneled subcutaneously above the right shoulder and secured above the scapula. The incisions were infiltrated with 1% lidocaine, and mice were placed in warm cages to recover from surgery. All mice were individually housed in a shoebox cage and transferred to a light (with 12-hour light/dark cycles) and temperature-controlled room in the animal facilities. Food and water were available ad libitum. The mice were given 7 days to recover from surgery before baseline mean arterial pressure (MAP), heart rate and locomotor activity were recorded for at least one week. Using isoflurane anesthesia, osmotic minipumps (Alzet, Durect, Cupertino, CA, USA) were implanted subcutaneously for 12–14 days to deliver either vehicle (saline) or Ang II at a rate of 400 ng/kg/min. WT and PPAR-*α* KO mice were also treated with the PPAR-*α* agonist fenofibrate at a dose of 145 mg/kg/day in corn oil, intragastrically (ig), three days prior to the implantation of the Ang II osmotic minipumps and throughout Ang II hypertension. Four groups of mice were used to record MAP, WT + Ang II (*n* = 9), and PPAR-*α* KO + Ang II (*n* = 8), WT + Ang II + fenofibrate (*n* = 9), PPAR-*α* KO + Ang II + fenofibrate (*n* = 9).

### 2.1. Analytical Methods

MAP data were collected at 500 Hz for 5 seconds each minute, from 3 PM until 10 AM (i.e., 19 h). The 3 PM to 6 PM period and the 6 AM to 10 AM period together (7 h) were analyzed as “day” MAP, and the 6 PM to 6 AM period (12 h) was “night.”

### 2.2. Plasma IL-6 Concentration

In separate groups of mice (*n* = 8 per group), blood samples were collected from WT, PPAR-*α* KO, WT + Ang II, PPAR-*α* KO + Ang II, WT + Ang II + fenofibrate, and PPAR-*α* KO + Ang II + fenofibrate to isolate plasma. Plasma IL-6 concentrations were measured by enzyme immunoassay (R&D Systems, Minneapolis, MN, USA) from terminal femoral artery blood samples obtained on day 12 of Ang II treatment. Animals from all groups were included on the same assay plate to control for interassay variability.

### 2.3. Western Blot

Whole kidney homogenized protein samples (50 *μ*g) were separated from the above mentioned groups (*n* = 4 per group) by sodium dodecyl sulfate-polyacrylamide gel electrophoresis on a 10% Tris-glycine gel, and proteins were transferred electrophoretically to a PVDF membrane. Nonspecific binding sites were blocked by incubating the blots overnight at 4°C in a Tris-NaCl buffer (TBS) containing 5% nonfat dry milk and 0.1% Tween 20. The primary antibodies used are rabbit CYP4A, CYP2J (1 : 2000; Santa Cruz, CA, USA), COX-2 (1 : 2000, Cayman chemicals, MI, USA), and ICAM-1 (1 : 1000 R & D, MN, USA). The blots were then washed in a TBS—0.1% Tween and incubated with the secondary antibody goat anti-rabbit 1 : 5000 and goat anti-mouse 1 : 10000 for *β*-actin, conjugated to horseradish peroxidase for 1 hour and washed. Detection was accomplished using enhanced chemiluminescence Western blotting, and band intensity was measured densitometrically, and the values were normalized to *β*-actin.

### 2.4. Renal MCP-1 Assay

Renal monocyte chemoattractant protein-1 (MCP-1) was also assessed in kidney homogenates using commercial available kit from R & D (MN), and values were normalized to mg protein (*n* = 5).

### 2.5. Statistical Analysis

Mean arterial pressure data are presented as means ± standard errors. Data were analyzed with a two-factor, repeated-measures ANOVA. Significant *F*-tests from the ANOVA at *P* < 0.05 was followed by post hoc comparisons using the Newman-Keuls multiple range test. A one-way ANOVA was used to analyze additional parameters with the Krusal-Wallis post hoc test. Significance was considered at *P* < 0.05.

## 3. Results

### 3.1. Ang II Hypertension

**Figure 1:**
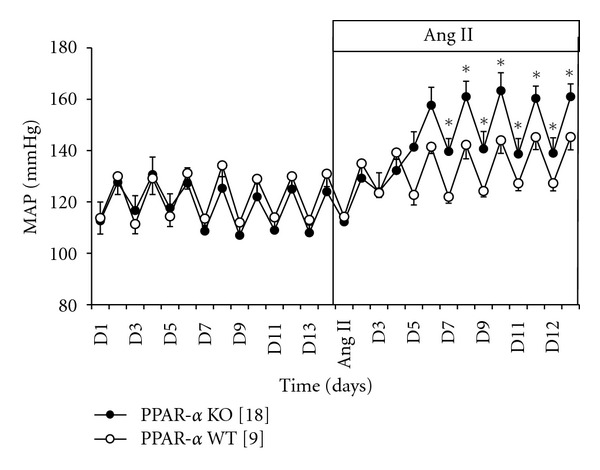
Mean arterial pressure of PPAR-*α* KO mice [18] (•) and PPAR-*α* WT mice [9] (o) during control days 1–13 and treatment of Ang II days 1–12. MAP was significantly higher in Ang II-treated PPAR-*α* KO versus Ang II-treated WT mice (*P < 0.05).

There was no significant differences in the baseline blood pressure between PPAR-*α* WT and KO mice. Day and nighttime averages for MAP were 113 ± 1 mm Hg and 131 ± 1 mm Hg, respectively, in the PPAR-*α* WT mice and 111 ± 2 and 127 ± 1 mm Hg, respectively, in the PPAR-*α* KO mice. During days and nights 5 through 12 of Ang II infusion (400 ng/kg/min), MAP significantly increased from baseline in the PPAR-*α* WT (125 ± 2 mm Hg (days) and 144 ± 1 mm Hg (nights)) and in PPAR-*α* KO mice (140 ± 2 mm Hg (days) and 161 ± 2 mmHg (nights)). The increase in MAP during days and nights 7–12 of Ang II infusion was significantly higher in PPAR-*α* KO than PPAR-*α* WT (Figure 1).

**Figure 2:**
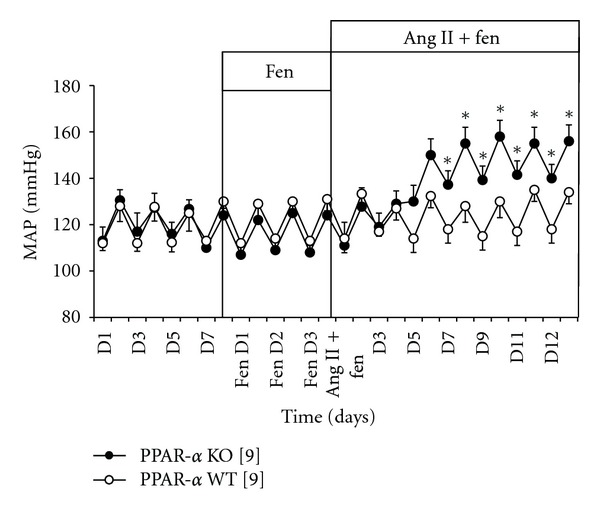
Mean arterial pressure of PPAR-*α* KO mice [18] (•) and PPAR-*α* WT mice [9] (o) during control days 1–7, days 1–3 of fenofibrate treatment, and days 1–12 of Ang II + fenofibrate treatment. MAP was significantly higher in KO + Ang II + Fen versus WT + Ang II + Fen (*P < 0.05).

 In a separate group of WT and KO mice, a three-day pretreatment with fenofibrate (145 mg/kg/day) alone did not significantly lower blood pressure in PPAR-*α* WT and PPAR-*α* KO mice (Figure 2). Fenofibrate significantly attenuated MAP during days 7 through 12 of Ang II hypertension in PPAR-*α* WT mice, 117 ± 6 mm Hg (days) and 132 ± 6 mm Hg (nights) when compared to PPAR-*α* WT + Ang II. Average MAP in PPAR-*α* KO mice during days and nights 7 through 12 of fenofibrate + Ang II treatment averages was 140 ± 7 mm Hg (days) and 158 ± 7 mm Hg (nights) (Figure 2).

**Figure 3:**
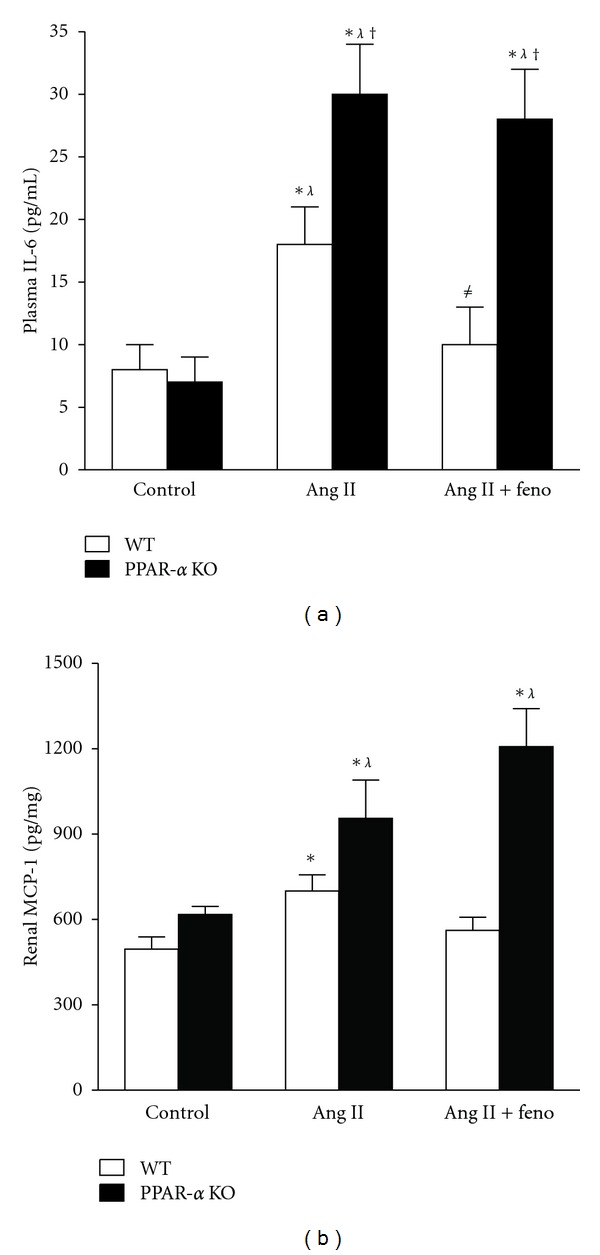
Plasma interleukin-6 levels (*n* = 8 per group) and renal MCP-1 levels (*n* = 5 per group) in control, Ang II-infused and Ang II-infused + fenofibrate-treated WT and PPAR-*α* KO mice. *indicates a significant increase versus control WT. *λ* indicates a significant increase versus control PPAR-*α* KO. ^†^indicates a significant increase versus Ang II-infused WT mice. ^*≠*^indicates a significant decrease versus Ang II-infused WT or PPAR-*α* KO mice.

 Plasma IL-6 levels were measured in control, Ang II-treated, Ang II-treated + fenofibrate WT, and PPAR-*α* KO mice. During control conditions, plasma IL-6 levels were similar between PPAR-*α* KO (7 ± 2 pg/mL) and PPAR-*α* WT (8 ± 2 pg/mL) mice. On day 12 of Ang II treatment, plasma IL-6 levels were significantly increased in PPAR-*α* KO (30 ± 4 pg/mL) and PPAR-*α* WT (18 ± 3 pg/mL) mice when compared to control conditions; however, this increase was much higher in Ang II-treated PPAR-*α* KO versus Ang II-treated WT mice. Fenofibrate significantly reduced plasma IL-6 in PPAR-*α* WT + Ang II mice (10 ± 3 pg/mL) when compared to PPAR-*α* WT + ANG II alone, whereas fenofibrate treatment failed to lower plasma IL-6 levels in PPAR-*α* KO + Ang II-treated mice (28 ± 4 pg/mL) (Figure 3).

Renal cortical MCP-1 was assessed as a marker of renal inflammation. There was no difference in basal MCP-1 levels between WT and PPAR-*α* KO mice. Infusion of Ang II significantly increased renal MCP-1 levels in both WT and PPAR-*α* KO mice, and fenofibrate treatment only decreased renal MCP-1 levels in WT mice (Figure 3).

**Figure 4:**
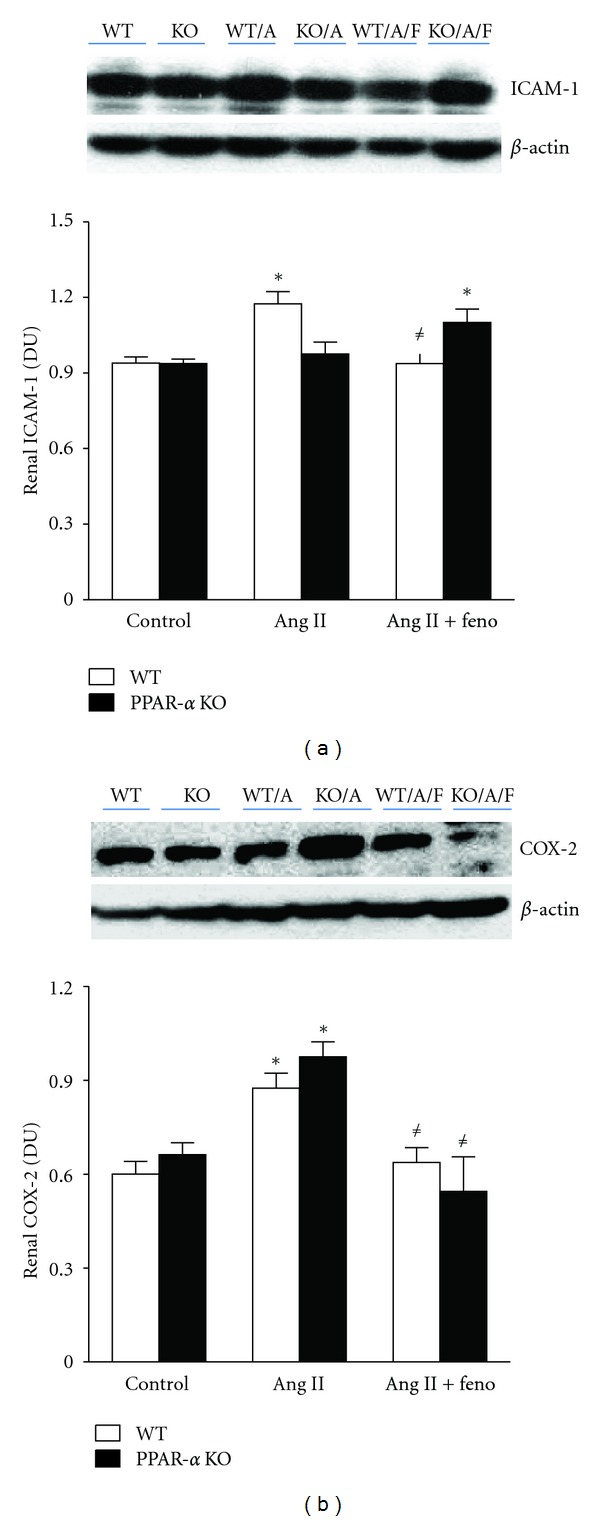
Renal ICAM-1 and COX-2 protein expression levels relative to *β*-actin as markers of renal inflammation in WT and PPAR*α* KO control, Ang II-infused and Ang II-infused + fenofibrate-treated mice. *indicates a significant increase versus control WT mice and ^#^indicates a significant increase versus Ang II-infused WT mice (*n* = 4 per group).

**Figure 5:**
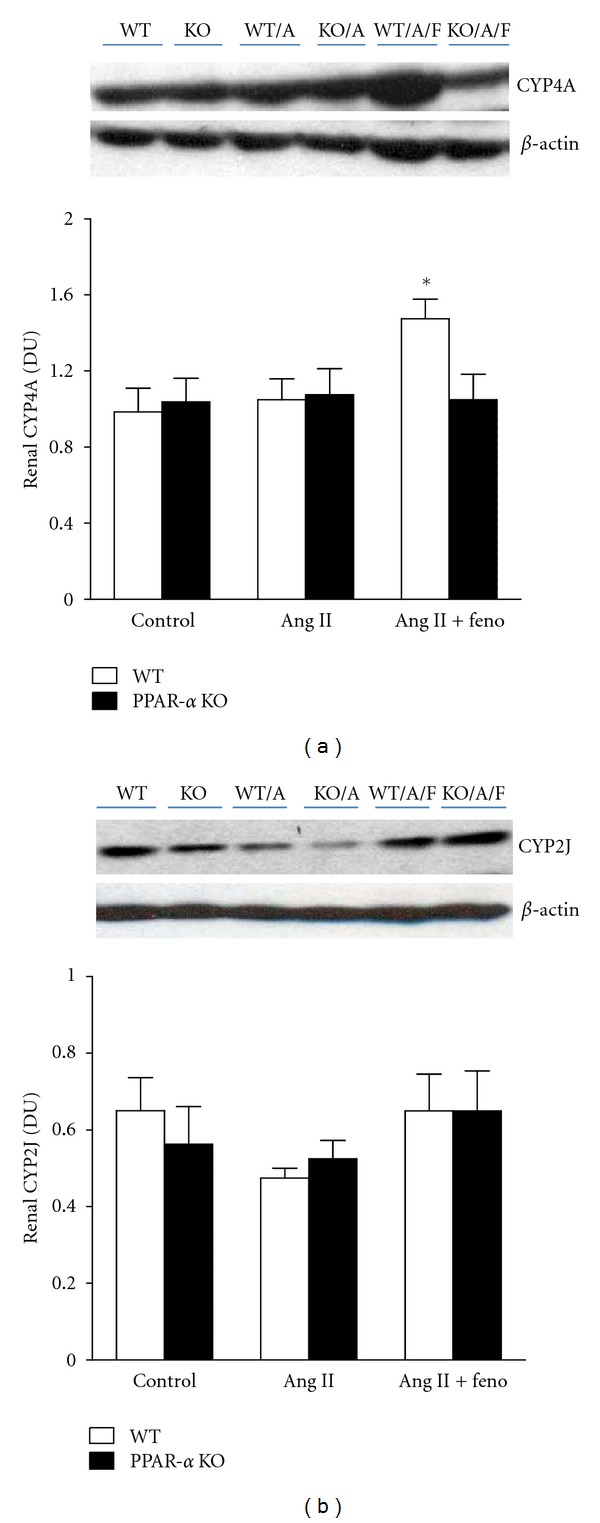
Renal CYP4A and CYP2J protein expression levels relative to *β*-actin in WT and PPAR*α* KO control, Ang II-infused and Ang II-infused + fenofibrate-treated mice. *indicates a significant increase versus control WT mice (*n* = 4 per group).

We also assessed protein expression of renal inflammatory markers in whole kidney homogenates of WT and PPAR-*α* KO mice treated with Ang II, with or without fenofibrate. Renal ICAM-1 expression was significantly higher in Ang II-treated WT mice compared to control WT or PPAR-*α* KO mice. Fenofibrate treatment significantly decreased the elevation in renal ICAM-1 expression only in Ang II-treated WT mice (Figure 4). Renal COX-2 expression was also significantly elevated in Ang II-treated WT and PPAR-*α* KO mice and fenofibrate treatment significantly reduced the elevation in renal COX-2 expression in both Ang II-treated WT and PPAR-*α* KO mice (Figure 4). Finally, we determined renal expression of CYP4A and CYP2J as indicative of 20-HETE and EETs production, respectively, in all groups. Our results indicate that fenofibrate treatment significantly increased renal CYP4A expression in Ang II-treated WT mice when compared to all other groups (Figure 5). Renal CYP2J expression was significantly decreased in Ang II-treated WT mice compared to WT control and fenofibrate treatment restored renal CYP2J expression in Ang II-treated WT mice to control WT (Figure 5).

## 4. Discussion

The main findings from this study are (1) PPAR-*α* KO mice have a significantly higher mean arterial pressure during days 7–12 of Ang II infusion, (2) fenofibrate attenuated MAP in WT + Ang II with no significant effect in PPAR-*α* KO + Ang II mice, (3) plasma IL-6 levels were significantly increased in PPAR-*α* KO when compared to WT + Ang II mice, (4) fenofibrate significantly reduced plasma IL-6 in WT + Ang II mice, (5) renal expression of CYP4A was significantly increased in WT + Ang II + fenofibrate mice, while MCP-1, ICAM-1, and COX-2 expression levels were elevated in WT + Ang II mice, and (6) fenofibrate treatment significantly decreased renal MCP-1, ICAM-1, and COX-2 expression in Ang II-treated WT mice.

 Previous studies demonstrate that the activation of PPAR-*α* causes a decrease in blood pressure in different models of hypertension. Fenofibrate has been shown to prevent the development of Ang II-dependent hypertension in mice [12]. The antihypertensive effect of fenofibrate was associated with a marked increase in renal CYP4A expression in Ang II-treated mice [12]. The study suggested that the upregulation of 20-HETE in renal tubules may contribute to the blood pressure-lowering effects of fenofibrate treatment in Ang II-dependent hypertension [12]. Clofibrate, a PPAR-*α* agonist, administration also lowers blood pressure and induces renal tubular 20-HETE production which reduces sodium retention in deoxycorticosterone (DOCA)-salt-hypertensive mice [11]. PPAR-*α* agonist WY14643 and clofibrate increases nitric oxide generation and promotes renal excretion of Na^+^ through reduced Na^+^-K^+^ ATPase activity in the proximal tubule [14, 35]. In the present study, we did not observe a decrease in renal CYP4A expression in mice treated with Ang II alone. A possible difference in the CYP4A expression results may involve the slow-pressor dose of Ang II (400 ng/kg/min) used in this study and a higher dose of Ang II (60 ng/min) used in a previous study [19]. However, in the current study, fenofibrate did cause a significant increase in renal CYP4A expression and attenuated MAP in WT + Ang II mice.

 The anti-inflammatory effects of PPAR-*α* activation have been shown in previous studies [36, 37]. Diep et al. demonstrated that fenofibrate caused a significant decrease in cardiac tissue proinflammatory markers including NF*κ*B and ICAM-1 [9]. In addition to the previous study, the PPAR-*α* activator, docosahexaenoic acid (DHA), abrogated the development of hypertension, decreased ICAM-1, VCAM-1, corrected structural abnormalities, and improved the endothelial dysfunction induced by Ang II [9, 18]. The present study demonstrates that anti-inflammatory mechanisms of PPAR-*α* activation also involves a decrease in renal MCP-1, renal expression of ICAM-1, COX-2, and plasma IL-6 during Ang II hypertension. The reduction in the renal expression of COX-2 in PPAR-*α* KO + Ang II + fenofibrate-treated mice may involve the suppression of inflammatory responses associated with the NF*κ*B pathway and appears to be independent of PPAR-*α*. The reduction of COX-2 renal expression in PPAR-*α* KO + Ang II + fenofibrate treated mice did not cause a reduction in MAP during Ang II hypertension. Future studies are needed to determine if the PPAR-*α* agonist mediated reduction in renal COX-2 expression is important for reducing blood pressure and renal damage.

The current study demonstrates that PPAR-*α* is necessary for the attenuation of plasma IL-6, an anti-inflammatory property that reduces blood pressure during Ang II-induced hypertension [24]. The results also indicate that the increase in plasma IL-6 corresponds to the significant increase in blood pressure in Ang II-treated PPAR-*α* KO mice. No significant changes were observed in plasma IL-6 of PPAR-*α* KO and WT during control conditions (Figure 3). The current study suggests that the activation of PPAR-*α* reduces mean arterial pressure during chronic Ang II-hypertension through an anti-inflammatory process that involves the significant reduction of plasma IL-6.

In summary, this study demonstrates that the activation of PPAR-*α* is important for reducing blood pressure during chronic Ang II hypertension. Our results suggest that the absence of PPAR-*α* exacerbates the elevation in MAP, plasma IL-6 levels and renal MCP-1 and COX-2 in Ang II-infused mice. We demonstrate that the blood pressure-lowering effect of a PPAR-*α* agonist during Ang II hypertension involves anti-inflammatory mechanisms and upregulation of the cytochrome P450 metabolites that influence renal vascular tone and tubular function. Future studies are needed to determine the relative contribution of the anti-inflammatory and direct renal actions of PPAR-*α* activation on lowering blood pressure.
